# Efficacy of SPECT over planar bone scan in the diagnosis of solitary vertebral lesions in patients with low back pain

**DOI:** 10.4103/0972-3919.72685

**Published:** 2010

**Authors:** Pushpalatha Sudhakar, Anshu Rajnish Sharma, Shanti M Bhushan, G Ranadhir, G Narsimuhulu, V.V.S. Prabhakar Rao

**Affiliations:** Department of Nuclear Medicine, NIMS, Mumbai, India; 1 Department of Nuclear Medicine, Tata Memorial Center (TMC), Mumbai, India; 2 Departments of Rheumatology, NIMS, Mumbai, India

**Keywords:** Bone SPECT, low backache, solitary vertebral lesion

## Abstract

**Background:**

The purpose of our study has been to evaluate the efficacy of single photon emission computed tomography (SPECT) over planar bone scan in identifying solitary vertebral lesions in patients with low backache and its ability to differentiate various pathologies according to the uptake pattern.

**Materials and Methods:**

The study included twenty patients out of whom six patients presented with known carcinoma and fourteen patients with low back pain. SPECT was done in all following planar skeletal survey. Benign and malignant lesions were identified according to the uptake pattern in vertebral elements, based on Gary F. Gates observations. Final diagnosis was obtained by means of biopsy or correlation with radiograph or computed tomography (CT) or magnetic resonance imaging (MRI), and / or follow up.

**Results:**

SPECT detected additional 30% of solitary vertebral lesions that were obscured on planar scan. Seven out of twenty were localized in anterior vertebral body and were diagnosed as benign ostophytes in six and osteoma in one substantiating the previous observations. Out of six cases of known carcinoma, three were having solitary metastases and showed posterior vertebral body uptake with pedicle involvement. SPECT could localize specific lesions as source of pain in eleven patients with low back pain (78%) and identified various etiologies including benign tumors (osteoid osteoma and osteoma), facet arthritis, discitis, transverse process fractures and spondylolysis.

**Conclusion:**

Our study highlighted the higher diagnostic value of SPECT over planar skeletal scintigraphy in localizing solitary vertebral lesions in low backache patients. Based on SPECT pattern, malignant and benign lesions could be differentiated in the given clinical context.

## INTRODUCTION

Low backache (LBA) is the frequently presenting complaint in everyday clinical practice. The common bone diseases that cause back pain include primary or metastatic tumors, fractures, infections, degenerative disc disease, facet arthritis, defects in the pars inter articularis and post operative pathologies.

LBA evaluation comprises different imaging modalities in which radionuclide bone scan plays an important role. In view of better anatomical localization with cross-sectional imaging that are comparable with that of computed tomography (CT) and magnetic resonance imaging (MRI), single photon emission computed tomography (SPECT) has become the first choice of imaging modality in LBA evaluation.[1]

## MATERIALS AND METHODS

Prospective analysis of bone SPECT was conducted in twenty patients who were referred for low back pain evaluation from various clinical departments. Eight out of 20 patients were women. Mean age was 25 years (range, 15 to 55 years) Skeletal scintigraphy was performed at 3 h following intravenous administration of 750 MBq of Tc 99m methylene diphosphonate (MDP) using dual detector gamma camera (Siemens E Cam) with low energy all purpose (LEAP) collimator. SPECT images were acquired in 180 degrees, 20 s per sample, using 128 × 128 matrix sizes. Butterworth filter (cut-off of 5.5 and order of 5) was applied for processing. Images were reconstructed in the transaxial, coronal and sagittal planes. Site of increased tracer uptake was localized either in vertebral body or in posterior vertebral arch. Tracer focus was identified at different vertebral arch components.

SPECT lesions were categorized as benign and malignant according to the pattern of lesion localization as summarized by Gary F. Gates.[2] SPECT patterns were compared with final diagnosis based on available imageological and histopathological data and clinical follow up.

## RESULTS

Twenty patients with LBA were evaluated prospectively with bone SPECT. Six patients with known carcinoma were referred from oncology department. Rest of the 14 were referred from orthopedics and rheumatology departments out of which four were having spondyloarthropathy and four had history of sports activity.

Planar bone scan was negative in six patients. SPECT showed 30% improved lesion detection as compared to planar bone scan. Three out of six lesions in carcinoma patients were established as metastases. Eleven out of fourteen lesions (78%) in nononcological referral patients were diagnosed with various specific pathologies as source of pain. SPECT patterns based on the site of tracer localization in different clinical conditions are detailed in Tables 1 and 2.

**Table 1:** The correlative data of SPECT patterns with final outcome in patients with known carcinoma (*n* = 6)

SPECT pattern/tracer localization	Number (*n*)	Final diagnosis		
		Radiograph	CT/MRI	Biopsy
Vertebral body with anterior extension	3	Osteophyte	-	-
Posterior vertebral body extending on to pedicle	2	-	-	Metastases
Isolated pedicle	1	-	-	Metastases

**Table 2:** The correlative data of SPECT patterns with final outcome in patients with low backache from non oncological referral (*n*=14)

SPECT pattern of tracer localization	Number (*n*)	Final diagnosis		
		Radiograph	CT/MRI	Biopsy
Vertebral body with anterior extension	3	Osteophyte	-	-
Vertebral body alone	1	-	Osteoma	-
Isolated pedicle	1	-	-	Osteoid osteoma
Facet joints (on the same horizontal plane of disc space just behind the pedicle on sagittal slice)	4	-	Facet Arthritis	-
Pars interarticularis (below the disc space and behind the posterior margin of vertebral body on sagittal slice)	2	Spondylolysis	-	-
Transverse process* / spinous process	2	-	Stress related fracture* /avulsion	-
Centered over disc space	1	-	Discitis	-

**Figure 1:**
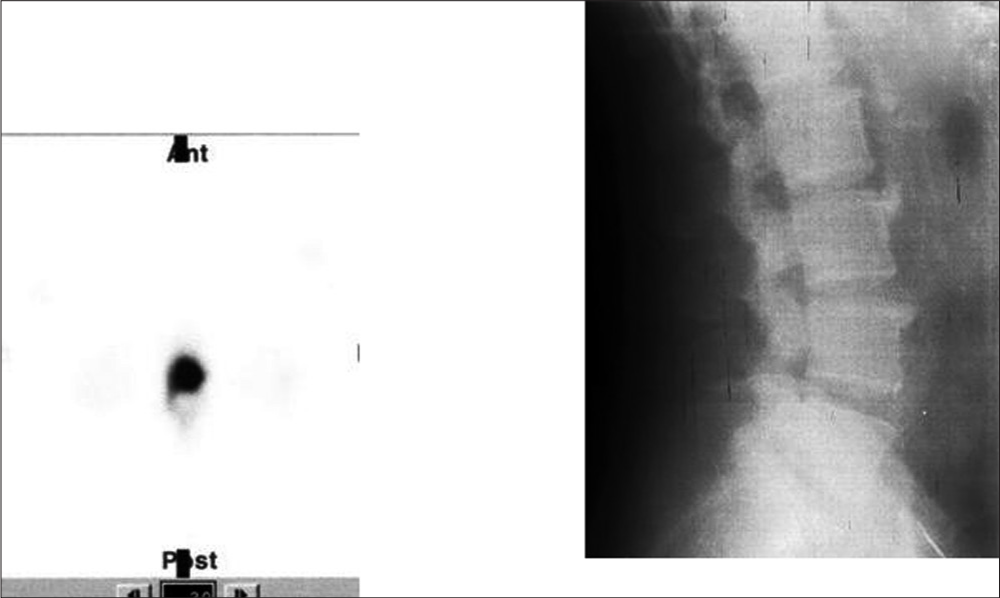
A case of vertebral osteophyte showing anterior extension of tracer uptake from the vertebral body. Radiograph confirms the diagnosis

Out of twenty, seven lesions were localized in vertebral body and were presumed to be benign in nature according to the SPCET pattern. Six were localized in body of the vertebra with anterior extension and were confirmed as osteophytes on radiographs [Figure 1]. One lesion localized in posterolateral aspect of vertebral body was diagnosed as benign osteoma on CT. SPECT was diagnostic in this case in which planar scan was normal [Figures 2a and b].

**Figure 2a:**
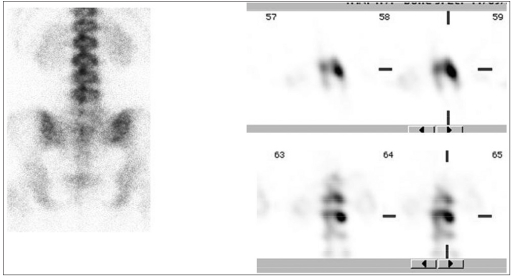
LBA patient with normal planar bone scan. SPECT localizing the solitary lesion in the vertebral body left laterally suggesting a benign pathology

**Figure 2b:**
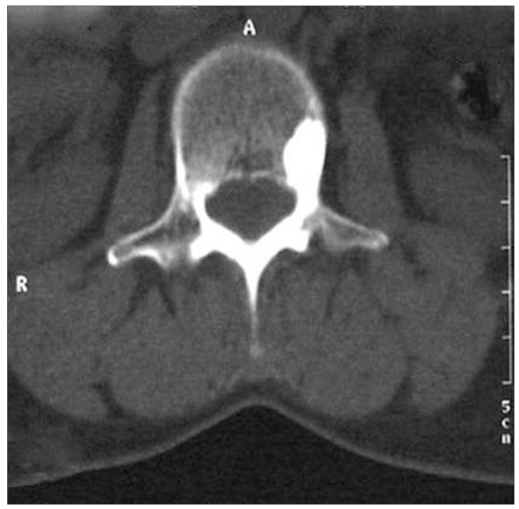
Further evaluation with CT showing hyper dense sclerotic lesion diagnosing benign osteoma

**Figure 3:**
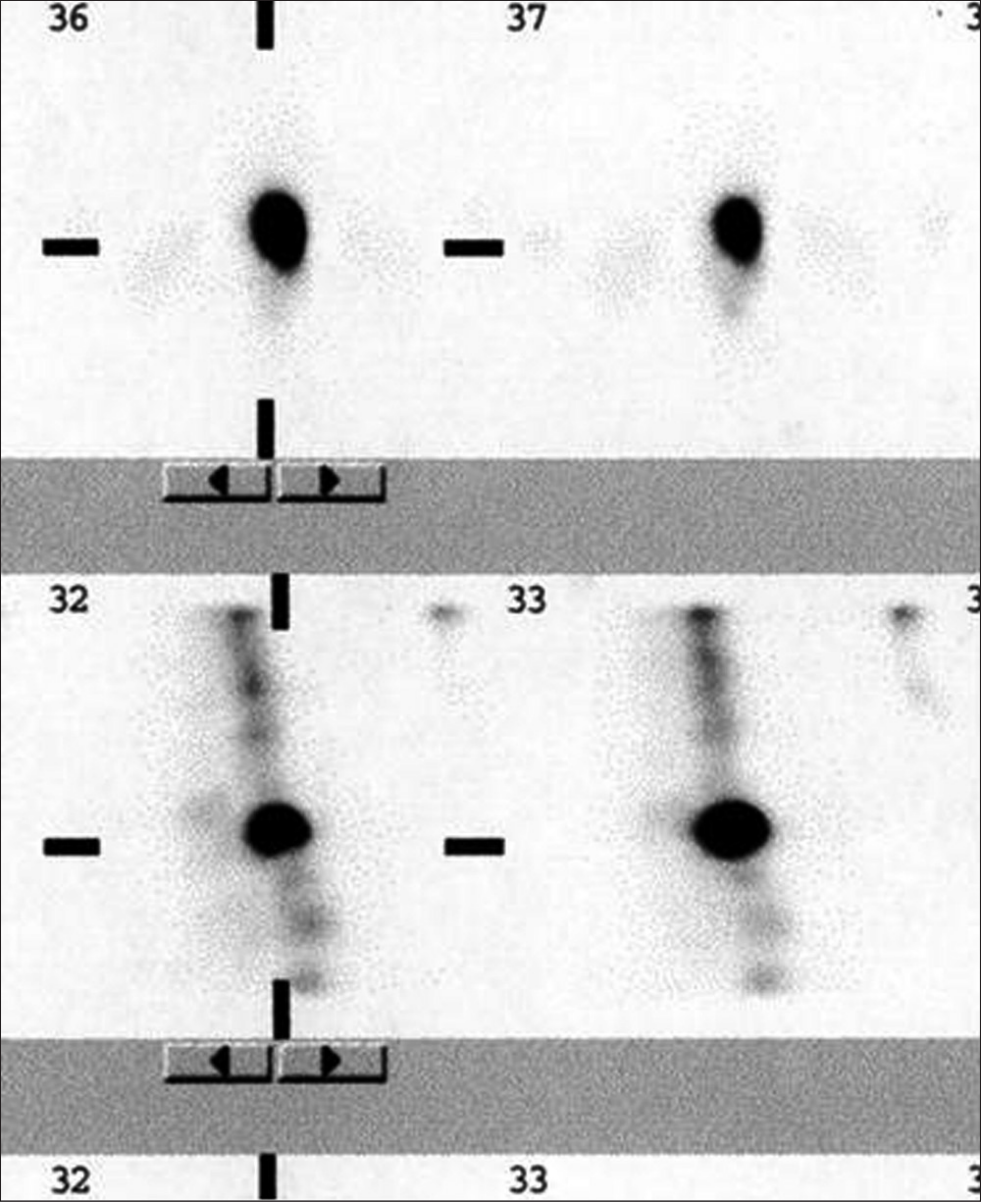
Solitary metastatic focus showing tracer uptake in the vertebral body along with left pedicle involvement

Out of three metastatic lesions, SPECT showed posterior vertebral body uptake with pedicle extension in two [Figure 3] and pedicle involvement alone in one lesion. In a young patient with LBA and equivocal radiograph, SPECT localized the lesion in L3 pedicle which was diagnosed as osteoid osteoma on CT with evidence of central sclerotic nidus [Figures 4a and b] Complete resection of the tumor relieved the patient from pain.

**Figure 4a:**
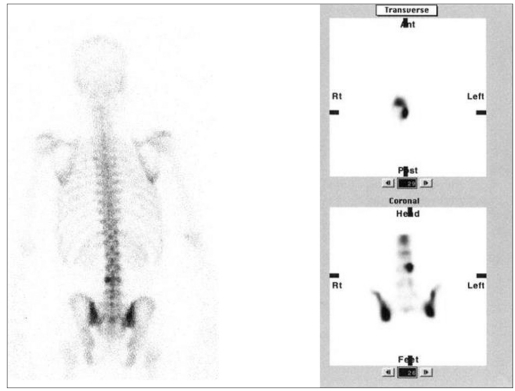
A case of vertebral osteoid osteoma in which SPECT was helpful in anatomic localization of the lesion in the left pedicle

**Figure 4b:**
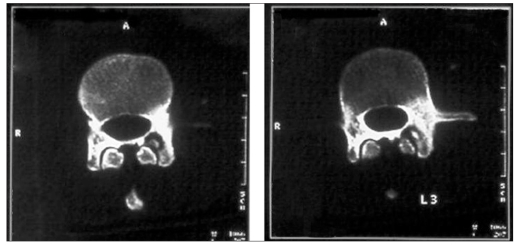
CT scan confirming osteoid osteoma in left pedicle with classical central sclerotic nidus

In patients with spondyloarthropathy, lesion was localized in the region of facet joints revealing facet arthritis as source of pain. Focal uptake over the region of pars interarticularis on sagital slices diagnosed spondylolysis in two athletic patients. Whereas foci over transverse process and spinous process detected fracture and avulsion injury respectively in the rest two athletes. In a single case with discitis, typical uptake of tracer centring over the disc was noted.

## DISCUSSION

Single photon emission computed tomography is more sensitive in diagnosing solitary vertebral lesions which are obscured on planar skeletal scintigraphy. SPECT allows 20% to 50% greater lesion detection than planar imaging.[3] Improved lesion detection with SPECT was noted in 30% of our study group which is in concordance with the previous observations.

SPECT has the advantage of providing anatomical localization of abnormal tracer focus in different components of vertebra that is essential to differentiate various spinal diseases which follow certain predictable patterns. The patterns of uptake in vertebrae on SPECT allow malignant disease to be distinguished from more benign pathology.

Isolated vertebral body lesions are usually benign. Uptake extending outward beyond the vertebral margin is usually seen with osteophytes. Apart from osteophytes, a simple osteoma was diagnosed in one of the vertebral body lesions in our study confirming the occurrence of benign pathology in the isolated vertebral body involvement.

SPECT has a diagnostic sensitivity of up to 93% in diagnosing solitary vertebral metastases.[4] Out of six patients with known malignancy in our study, metastasis was diagnosed in three. All of them showed characteristic pattern of uptake as depicted by Gary F Gates *i.e*. predominant involvement of posterior vertebral body along with pedicle involvement. Metastasis was ruled out in the rest three which showed benign osteophyte uptake pattern. Agra *et al* reported that 99% of the lesions in their study involved the posterior portion of vertebral body and among them 60% showed simultaneous pedicle involvement[5] Hematogenous spread through posteriorly located basivertebral veins was thought to be the cause of posterior predilection of vertebral body with pedicle involvement.

SPECT gives better anatomical localization of vertebral arch lesions than planar scan particularly in apophyseal joints.[6] Posterior arch lesions are usually benign, frequently osteoarthritc conditions, occasionally fractures, inflammatory diseases and osteoid osteomas etc.[7]

Diagnosis of facet arthritis as a cause of LBA is helpful in injection therapy. The high negative predictive value of SPECT is helpful in this aspect. Surgical outcome can be predicted on the basis of metabolic activity of spondylolysis by SPECT. Patients with positive SPECT show better post operative outcome than those with negative study.[8]

**Figure 5:**
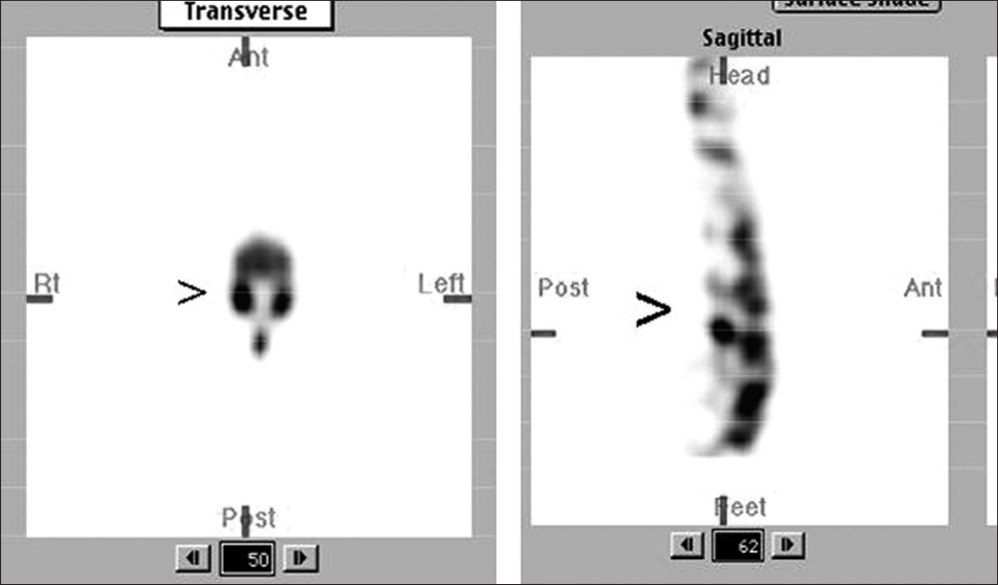
Sagittal slices with focal tracer uptake on the same horizontal plane of disc space just behind the pedicle: classical of facet arthritis

**Figure 6:**
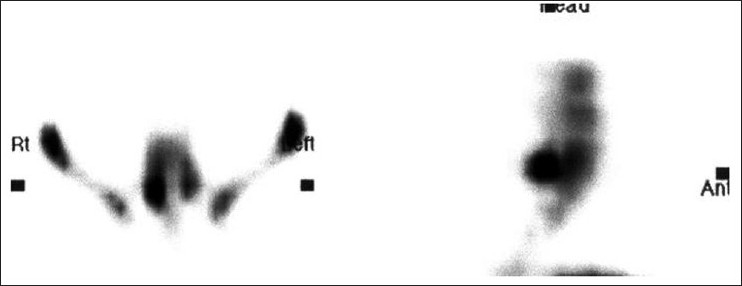
Focal uptake below the disc space and behind the posterior margin of vertebral body corresponding to the site of pars interarticularis is s/o spondylolysis

Oblique or sagittal sections of SPECT constructed slightly off mid line toward affected side will differentiate facet arthritis from spondylolysis. Focal tracer uptake on the same horizontal plane of disc space just behind the pedicle is suggestive of facet joint arthritis [Figure 5] Focal uptake below the disc space and behind the posterior margin of vertebral body corresponding to the site of pars interarticularis is indicative of spondylolysis [Figure 6]. With this pattern of tracer localization, we could precisely identify facet arthritis in four and spondylolysis in two patients with LBA.

10% of osteoid osteomas occur in spine, the common locations are lamina, pedicle, and articular facets in the order of frequency. Precise siting is often critical to surgery. Accurate anatomical localization in vertebral components is best achieved with SPECT.[9] Addition of SPECT helped in precise localization of osteoid osteoma of the pedicle as a source of low back pain in one of our patient. Complete pain relief was achieved following surgical excision.

**Figure 7:**
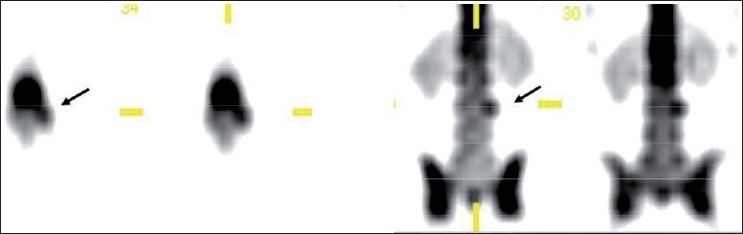
L3 vertebral left transverse process fracture in an athletic patient

Increased tracer uptake in spinous process or transverse process is commonly due to fractures, and rarely due to neoplasms, whereas laminar abnormality can be secondary to fracture or osteoid osteoma. In our study, SPECT was diagnostic in a case of athletic patient revealing transverse process fracture as the source of pain [Figure 7] Rarely, rotational movements may lead to avulsion / traction injuries of the multifidus muscles of the lumbar spine, presenting as increased uptake in the spinous process.[10]

**Figure 8:**
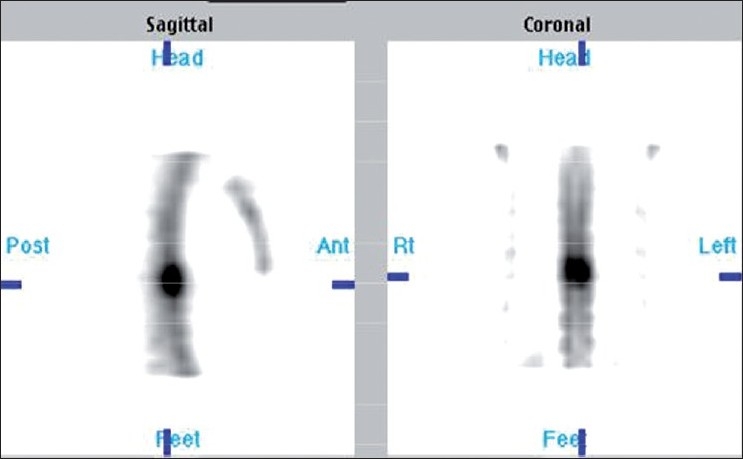
Discitis showing typical vertical ovoid configuration of tracer concentration

Discitis is an infection or inflammatory condition involving intervertebral disc. Bone scan with SPECT is more sensitive than other imaging modalities.[11] Tracer uptake often has vertical ovoid configuration rather than a horizontal pattern which is seen in compression fracture [Figure 8].

## CONCLUSION

Our study highlighted the higher sensitivity of SPECT over planar scan in detecting and localizing solitary vertebral lesions with 30% improved lesion detection. The pattern of vertebral uptake in different clinical conditions was in concordance with that specified by previous authors. Addition of SPECT to planar bone scan is highly recommended in patients with low backache to localize the source of pain.
